# Adjunctive dry needling reduces spasticity and improves functional outcomes in children with cerebral palsy: a single-blind randomized controlled trial

**DOI:** 10.3389/fped.2026.1865049

**Published:** 2026-08-27

**Authors:** Nouf H. Alkhamees, Osama R. Abdelraouf, Mohamed A. Abdel Ghafar, Zizi M. Ibrahim, Eman M. Harraz, Mohamed K. Seyam, Walaa E. Morsy, Mohamed M. Ahmed

**Affiliations:** 1 Department of Rehabilitation Sciences, College of Health and Rehabilitation Sciences, Princess Nourah bint Abdulrahman University, Riyadh, Saudi Arabia; 2 Physical Therapy Program, Batterjee Medical College, Jeddah, Saudi Arabia; 3 Department of Physical Medicine, Rheumatology and Rehabilitation, Faculty of Medicine, Mansoura University, Dakahlia, Egypt; 4 Department of Physical Therapy and Health Rehabilitation, College of Applied Medical Sciences, Majmaah University, Majmaah, Saudi Arabia; 5 Physical Therapy Department, College of Nursing and Health Sciences, Jazan University, Jazan, Saudi Arabia; 6 Department of Pediatrics, Faculty of Physical Therapy, Cairo University, Cairo, Egypt; 7 Department of Basic Sciences in Physical Therapy, Faculty of Physical Therapy, Beni-Suef University, Beni-Suef, Egypt

**Keywords:** dry needling, functional mobility, gross motor function, neuromuscular rehabilitation, spastic cerebral palsy

## Abstract

**Background:**

Spastic cerebral palsy is the most prevalent motor disability in childhood, and the alleviation of spasticity has been a primary rehabilitation objective with the view of improving the outcomes of functional recovery. Dry needling (DN) has demonstrated some efficacy in adult neurology, but controlled evidence is limited in children. This study tested the hypothesis that incorporating DN into traditional physical therapy is effective in improving spasticity, gross motor functions, and functional mobility in ambulant children with spastic cerebral palsy.

**Methods:**

Forty-eight children with spastic diplegia cerebral palsy, ages 6–12 years, were randomly allocated to either a DN group (DN plus traditional therapy) or a traditional rehabilitation (TR) group (traditional therapy alone). Interventions lasted 12 weeks. Outcome measures included the Modified Ashworth Scale (MAS), Gross Motor Function Measure (GMFM-88; dimensions D and E), and Timed Up and Go (TUG) test.

**Results:**

MANOVA analyses showed that both groups significantly improved across all outcomes (*p* < 0.05). The DN group showed greater improvements in MAS (mean change: −0.5 vs. −0.2), GMFM-88 (mean change: +10.2 s vs. +5.9 s), and TUG (mean change: −4.2 s vs. −1.4 s). Spasticity, gross motor, and functional mobility outcomes favored the DN group (*p* = 0.02, *p* = 0.02, and *p* = 0.002). One small adverse event was observed with no treatment discontinuations.

**Conclusion:**

Dry needling in combination with traditional physical therapy was associated with greater improvements in spasticity, motor function, and mobility than traditional therapy alone. DN was tolerable and could be a safe and effective additional intervention for ambulatory children with mild-to-moderate spastic cerebral palsy.

**Clinical Trial Registration**: ClinicalTrials.gov, identifier NCT06856473.

## 1 Introduction

Cerebral palsy (CP) is the most prevalent motor disability among children worldwide with the occurrence of one in 500 live births (1, 2). CP results from non-progressive disturbances of the developing brain, leading to permanent disabilities, which adversely affect movement, posture, and coordination (3).

The condition occurs in various forms depending on the level of severity and is categorized based on the type of motor and patterns of distribution. Most cases of cerebral palsy belong to spastic subtype because it accounts for approximately 80% of cases with this condition, thus, making the muscles of the patient become rigid and the reflexes become exaggerated (4). In spastic cerebral palsy, damage to motor control pathways, including the corticospinal tract, disrupts normal descending inhibition and increases reflex excitability, resulting in spasticity (1). Accordingly, spasticity should be viewed as a manifestation of broader central nervous system injury rather than being attributed solely to corticospinal dysfunction (4, 5). The persistence of spasticity causes muscle contractures, skeletal deformities, and pain over time translating to the inability of children to perform daily activities and walk independently (6). Spastic diplegia is one of the most prevalent patterns of spastic forms, which is mostly involved in the lower extremities and is usually related to gait and mobility impairments (7).

Spasticity in children with CP typically needs multidisciplinary therapy, which may include physical therapy, medication and in some instances surgical intervention to improve motor control and mobility (8). Some of the treatment strategies that can be employed in children with spasticity are oral antispastic drugs such as baclofen and tizanidine, intrathecal baclofen pumps, and botulinum toxin injections. These treatments are able to minimize muscle tone and are usually short-lived and with side effects including weakness and sedation (8, 9). Selective dorsal rhizotomy is a surgery, which can help give long-term results, though it is important to select the candidates carefully considering potential surgical complications (10). Scientists continue to research new non-invasive interventions, which can offer long-term spasticity reduction and improved functional outcome without medical risks (11).

Dry needling (DN) is a new neuromuscular intervention that can be implemented in neurorehabilitation. It is done by inserting fine monofilament needles into the hypertonic and myofascial areas without any drug addition, which helps patients to have a normal muscle activity and become less tense in the spastic muscles (12). DN is based on the principles of neurophysiology as opposed to traditional acupuncture techniques which are based on local mechanic reactions and reflex regulation. The treatment acts through three primary processes, which are activation of the sensory nerve endings and muscle spindles, the normalization of endplates, and inhibition of spinal and supraspinal reflexes (12, 13). Research on DN for neurological conditions is still in its early stages. Studies involving stroke and spinal cord injury patients have shown that DN treatment results in decreased spasticity according to the Modified Ashworth Scale, with improvements in mobility and reduction in pain symptoms (14–16). The treatment approach influences both local and central control systems of motor function, resulting in improved outcomes when patients receive DN therapy alongside their standard physical therapy program (17).

Evidence from adult dry needling research should not be generalized to children, whose developing nervous systems and muscle properties differ significantly. Unfortunately, DN research in pediatric CP populations remains scarce. However, only a few studies exploring the use of DN to manage spasticity in children with CP have reported promising reductions in resistance to passive movement following DN in children with CP (17, 18), but large-scale controlled trials are still lacking. Therefore, this study aims to evaluate the effects of DN on spasticity, gross motor function, and functional mobility in children with CP. By examining these outcomes, we aim to determine whether DN can serve as an effective adjunct therapy to improve motor abilities and reduce spasticity-related disability in this population.

## 2 Materials and methods

### 2.1 Study design and participants

This is a multicenter single-blinded (assessor-only), randomized controlled clinical trial. The Ethical Committee of Batterjee Medical College (RES-2025-0049) reviewed and approved the study procedures in accordance with the latest version of the Declaration of Helsinki. Additionally, the study was registered prospectively on ClinicalTrials.gov on February 23, 2025, under the ID NCT06856473. The parents and caregiver signed a written assent form after being made aware of the research procedures, risks, and benefits.

All study participants were recruited from four local pediatric rehabilitation centers in Jeddah, Saudi Arabia, to ensure broader recruitment. These centers were outpatient rehabilitation facilities that provide routine physiotherapy services for children with cerebral palsy and related developmental motor disorders. To maintain consistency, the same recruitment criteria and study procedures were applied across all sites.

A total of 70 children were screened for eligibility. Fifty-five children from both sexes were eligible according to the following criteria: age range from 6 to 12 years, with a confirmed diagnosis of spastic diplegic cerebral palsy by their doctor, classification within Gross Motor Function Classification System (GMFCS) Levels II–III; lower-limb spasticity ranging from 1+ to 2 according to Modified Ashworth Scale (MAS), able to stand and walk and capable of understanding and following instructions. Fixed contractures or deformities in lower extremities, a history of orthopedic surgery on the lower limbs, dorsal rhizotomy, botulinum toxin injection in the lower limb within the last 6 months or planning to have one during the study period were all exclusion criteria.

The study used a stratified random sampling method to allocate the participants into two groups (DN and TR groups). The stratification was made to control certain variables especially sex, and to ensure a balanced and representative sample. In each stratum, the participants were allocated in blocks of permuted blocks with four participants with a computer-generated random sequence. In order to preserve allocation concealment, the assignments of the participants were registered in non-transparent, sealed, and sequentially numbered envelopes, which reduced the possibility of selection bias. They were stored in these envelopes which were opened only during group allocation. The opening of the envelopes was carried out by an independent third party, which was not involved in the recruitment of the participants or the intervention process, which further ensured the integrity of the randomization process.

### 2.2 Sample size calculation

G*Power software (Heinrich-Heine-Universität Düsseldorf, Düsseldorf, Germany) was used to calculate sample size, with an alpha level of 0.05, a power of 80%, and an effect size of 0.5 being the values used, which was also the result of previous research on spasticity in children with cerebral palsy (19, 20). A total sample size of 45 children was deemed appropriate for the study. An anticipated 15% dropout rate was taken into account when determining the number of children to be recruited in both groups.

### 2.3 Evaluation procedures

#### 2.3.1 Spasticity score assessment

The Modified Ashwarth Scale (MAS) measures spasticity on a 6-point ordinal scale from 0 to 4, with an additional 1 + category. Minimum Value (0): No increase in muscle tone (better outcome). Maximum Value (4): Affected part(s) rigid in flexion or extension (worse outcome). Higher scores indicate greater spasticity, representing a worse outcome (21). Spasticity in hamstrings, adductors and calf muscles were assessed before and after the treatment. The child lay in the supine position, with head in the midline position to avoid the tonic-asymmetric reflex possibly causing increased spasticity.

#### 2.3.2 Gross motor function assessment

The motor performance changes were assessed using the Gross Motor Function Measure (GMFM). The GMFM-88 is formed of five dimensions (A–E), assessed on a four point ordinal scale 0 = does not initiate, 1 = initiates, 2 = partially completes, and 3 = completes the activity. The dimension scores are transformed into percentages and higher percentages indicate higher functional ability and lower percentages show more severe restrictions. GMFM has high inter-rater reliability and coefficients have been reported as high as 0.99 which attests to high measurement stability. Its construct validity has been proven by high correlation to known indicators of gross motor functioning, and it can be stated to be suitable in measuring children with motor impairment. This research involved the use of the questions of Dimension D (Standing) and Dimension E (Walking, Running, and Jumping) which were assessed before and after the therapeutic intervention after the standard testing procedures (22, 23).

#### 2.3.3 Functional ability Assessment

The time up and go test (TUG) is used to determine the time (in seconds) that it takes to stand up from a chair with armrests, walk 3 m, turn around, walk back to the chair, and to sit down. Two trials were undertaken by each of the participants and the results of both trials averaged. Minimum Value (Reduced time in seconds): Faster time of completion is suggested to be more mobile and functional. Maximum Value (higher time in seconds): Slower completion time indicates impaired mobility and a higher risk of falls (worse outcome). The test is a valid and reliable assessment of balance in normally developing children and those with disabilities (24).

All outcome measures were evaluated at baseline (pre-intervention) and after 12 weeks of intervention (post-intervention). The evaluation processes were carried out by a certified physiotherapist with more than ten years of experience in pediatric assessment and rehabilitation; he/she did not know how the participant was allocated. The authors provided a thorough explanation and oversight of a unified evaluation plan.

### 2.4 Intervention

The standardization of the delivery of physical therapy interventions was achieved through an exercise manual that provided the main elements of each session, dosing, exercise frequency, progression criteria, and pre-established equivalent forms of exercises across all centers. All treating therapists received centralized online training to ensure competency during the study intervention period. The intervention was delivered using electronic Case Report Forms (eCRFs) to document exercises performed, sets, repetitions, load, and intensity. A standard fidelity checklist was used to measure treatment adherence, which was assessed through periodic observations or by recording sessions after parents’ approval. The site-specific customizations were limited to options that were *a priori* approved, ensuring that the therapeutic content remained as intended.

Moreover, all dry needling interventions in this study were carried out according to a unified, standardized protocol so that there is methodological consistency across all participating centers. The certified therapists at each site received training. They strictly followed the same procedures and guidelines, including the choice of needle, insertion methods, targeted musculature, treatment dosage, safety measures, and documentation. No other physical agent modalities were applied in either group; the intervention was limited to the standardized traditional physical therapy program, with dry needling administered only to the study group as an adjunct intervention.

#### 2.4.1 Traditional physical therapy

Both the control group and the study group underwent one hour session of comprehensive conventional physical therapy, three sessions per week, for 12 weeks. Therapeutic objectives included improvement of independence and reducing the burden on caregivers. Based on neurodevelopmental principles, the intervention plan involved techniques that help to enhance muscle flexibility in muscle groups typically involved in spasticity, strengthen weakened muscles, develop postural stability, stimulate proprioception, and improve gait velocity. Functional task training, including sit-to-stand exercises and transfers, aimed at lower limb loading and the integration of activities of daily living (ADLs) (25). Written home exercise programs that promoted daily PROM, positional management, and task-specific activities to improve carryover benefits were provided to caregivers (26).

#### 2.4.2 Dry needling therapy

In addition to the standard program, children in the study group received dry needling targeting the hamstrings, adductors, and gastrocnemius muscles once a week for 12 weeks. During the intervention, three muscles in each leg were treated on alternate weeks, resulting in a total of six sessions per leg. The protocol was adopted from previous literature, which generally supports multiple needle insertions with 4–8 needles per session unless indicated by specific clinical reasoning (27). As there are no clear guidelines on the maximum number of dry needling points per session for the pediatric population, the authors chose a cautious approach to needle numbers to ensure safe tolerance to the procedure. The DN intervention was applied first during the session to facilitate the benefits of conventional physiotherapy (18, 27).

Certified physical therapists in DN, with at least five years of experience in pediatric rehabilitation, administered the treatment program while being blinded to the study's objectives and group allocation. The children were positioned in a way that provided safe and comfortable access to all muscles: the hamstrings and the gastrocnemius in the prone position, and the adductors in the hook-lying position with slight hip abduction (28). During the DN application, sterile technique was applied, which included application of single-use sterile needles, proper skin preparation, wearing of gloves, and controlled disposal of sharps in accordance with the national guidelines.

Two minutes before dry needle administration, Lidocaine spray was applied to the targeted muscle to alleviate pain during the skin penetration (29). Latent myofascial trigger points were detected by palpating the taut muscle bands, then monofilament needles, which were 0.16 mm in diameter and 25 mm in length, were slowly inserted perpendicular to the targeted points. The technique adopted was intentionally conservative, keeping the needle *in situ* for 5 min (18), while local twitch reactions were not elicited to ensure comfort and reduce post-needling pain in this spastic pediatric group (19). Light compression on each site was done several seconds after removing the needle to alleviate pain and lessen blood loss. A 20-min rest was allowed following DN to reduce soreness, maximize muscle relaxation, and improve treatment outcomes (30).

The safety and tolerability of dry needling were closely monitored throughout the intervention period. Any adverse events, including bruising, bleeding, soreness, or other unwanted reactions, were recorded. In addition, pain responses during and after the session was observed to ensure the procedure was well tolerated by the children.

### 2.5 Statistical analysis

All per-protocol analyses were performed using IBM SPSS Statistics 25 (IBM Corp., Armonk, NY, USA). The Shapiro–Wilk and Levene tests were used to assess data distribution, identify outliers, and assess the homogeneity of variances. The assumption of normality and homogeneity was met, and parametric tests were used. Independent *t*-tests were used to compare demographic and clinical variables, and a Chi-square test was used to compare sex distributions. To examine the changes in spasticity, gross motor function, and functional mobility, a 2 (group: DN vs. TR) × 2 (time: pre- vs. post-intervention) mixed-model multivariate analysis of variance (MANOVA) was conducted, filled with the following dependent variables: MAS scores, GMFM, and TUG performance. When a strong multivariate group × time interaction was observed, the 2 × 2 mixed-model ANOVAs were followed up separately on the outcomes to determine which variables contributed to the multivariate effect. Notable interactions in the univariate models were further investigated using simple effects analyses (within-group pre-post and between-group comparisons at each time point), and Bonferroni-adjusted alpha was used to control for multiple comparisons. Effect sizes were calculated using partial eta squared to indicate the practical relevance of the findings. The statistically significant level was determined as *p* < 0.05, two-tailed.

## 3 Results

**Figure 1:**
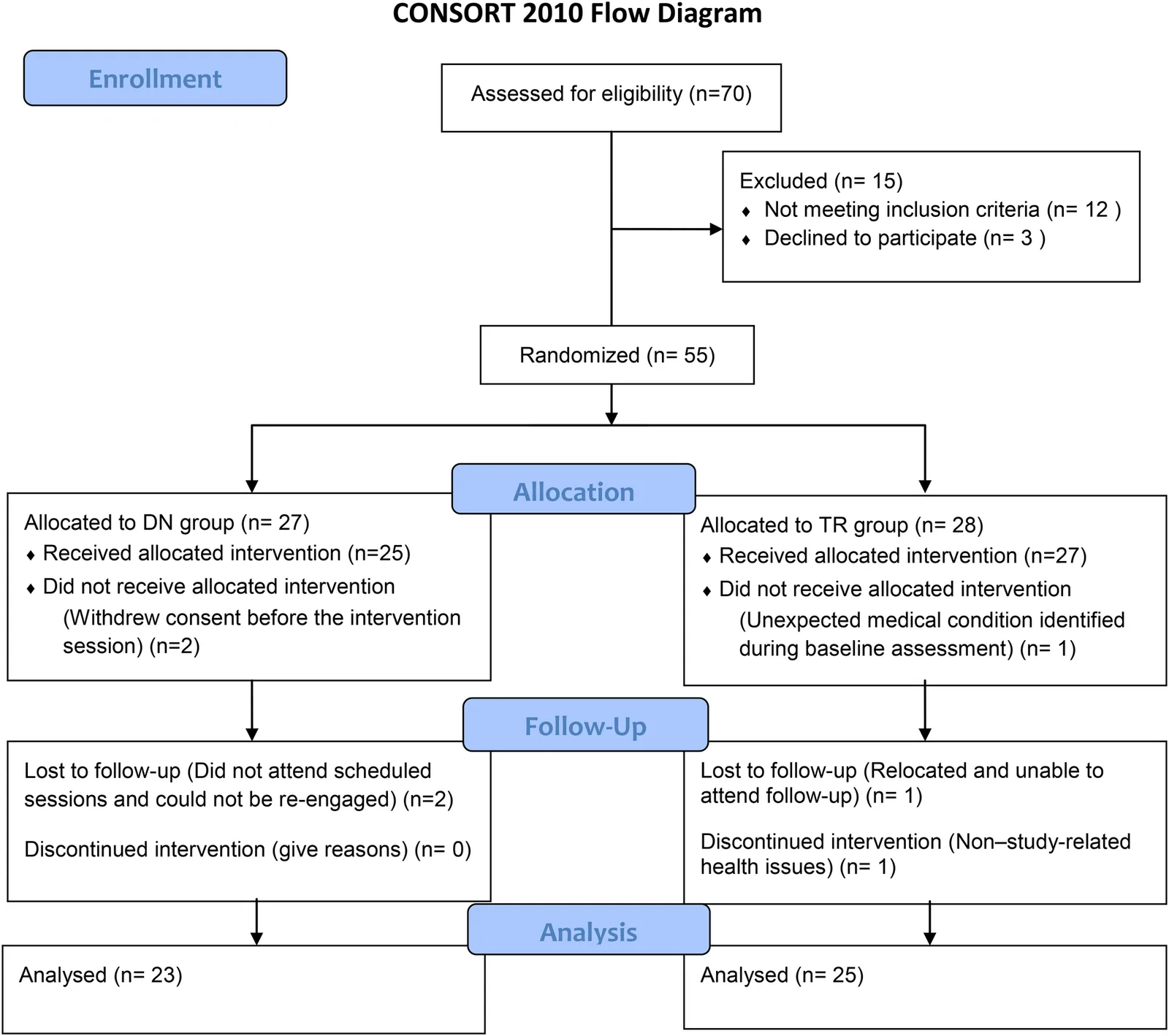
The flowchart of the whole study.

The analysis involved forty-eight children with spastic cerebral palsy, of whom 23 were given DN plus traditional physical therapy, and the other 25 received traditional physical therapy alone. The study flowchart is presented in Figure 1.

No significant differences were observed between the two groups in age, height, weight, and baseline spasticity (*p* > 0.05). Furthermore, there was no significant difference in gender distribution using Chi-square analysis (DN: 12 boys, 11 girls; TR: 13 boys, 12 girls (*p* = 0.82).

The mixed MANOVA analysis revealed a significant group × time interaction [Wilks’ Lambda = 0.68, *F* (3,44) = 6.9, *p* < 0.001], indicating that the DN and TR groups exhibited different patterns of change throughout the study. The combined outcome set showed a major group × time interaction, so we performed additional tests for each outcome. The subsequent univariate tests showed significant group-by-time interactions for MAS, GMFM, and TUG at all *p*-values <0.05. The research data showed that both groups achieved better results during the 12-week study, yet the DN group demonstrated larger improvements throughout the entire period.

### 3.1 Between-group Comparisons

At baseline, no significant differences were observed between groups in MAS scores (DN: 1.7 ± 0.3, TR: 1.6 ± 0.2, *p* = 0.18, *η_p_*^2^ = 0.005), GMFM-88 scores (DN: 45.6.8% ± 6.8, TR: 44.8% ± 7.3, *p* = 0.69, *η_p_*^2^ = 0.003), or TUG test times (DN: 15.4 ± 2.3 s, TR: 15.1 ± 2.5 s, *p* = 0.66, *η_p_*^2^ = 0.002). After 12 weeks, MANOVA revealed significant differences between groups, favoring the DN group across all outcomes (*p* < 0.01). The DN group showed a greater reduction in spasticity (MAS post: 1.2 ± 0.2 vs. TR post: 1.4 ± 0.4, *p* = 0.02, *η_p_*^2^ = 0.11), improvement in gross motor function (GMFM-88 post: 55.8% ± 7.1 vs. TR post: 50.7% ± 8.2, *p* = 0.02, *η_p_*^2^ = 0.09), and enhanced functional mobility (TUG post: 11.9 ± 1.8 s vs. TR post: 13.7 ± 2.1 s, *p* = 0.04, *η_p_*^2^ = 0.11) (Table 1).

**Table 1:** Between-group and within-group comparisons of outcome measures.

Outcome measure	Group	Pre-intervention (Mean ± SD)	Post-intervention (Mean ± SD)	Within-group *p*-value	Within-group MD (95% CI)	Within-group *η_p_*^2^
MAS (Score)	DN	1.7 ± 0.3	1.2 ± 0.2	<0.001*	−0.5 (−0.64 to −0.35)	0.12
	TR	1.6 ± 0.2	1.4 ± 0.4	0.03*	−0.2 (−0.38 to −0.01)	0.07
	Between-group *p*-value	0.18	0.02*			
	Between-group MD (95% CI)	0.1 (−0.29 to 0.04)	−0.2 (−0.38 to −0.01)			
	Between-group *η_p_*^2^	0.005	0.11			
GMFM-88 (%)	DN	45.6 ± 6.8	55.8 ± 7.1	<0.001*	10.2 (6.21 to 14.18)	0.16
	TR	44.8 ± 7.3	50.7 ± 8.2	0.01*	5.9 (1.38 to 10.41)	0.09
	Between-group *p*-value	0.69	0.02*			
	Between-group MD (95% CI)	−0.8 (−4.92 to 3.32)	−5.1 (−9.95 to −0.64)			
	Between-group *η_p_*^2^	0.003	0.09			
TUG (seconds)	DN	15.4 ± 2.3	11.9 ± 1.8	<0.001*	−4.2 (−5.41 to −3)	0.14
	TR	15.1 ± 2.5	13.7 ± 2.1	0.04*	−1.4 (−2.74 to – 0.05)	0.06
	Between-group *p*-value	0.66	0.002*			
	Between-group MD (95% CI)	−0.3 (−1.69 to 1.09)	1.8 (0.66 to 2.93)			
	Between-group *η_p_*^2^	0.002	0.11			

MAS, modified ashworth scale; GMFM-88, gross motor function measure; TUG, timed up and go test; MD, mean difference; CI, confidence interval, *η_p_*^2^, partial eta squared. All values are mean ± SD (standard deviation).

* *p*-value < 0.05 indicates a significant difference.

### 3.2 Within-group comparisons

Both the DN and the TR groups showed statistically significant improvements post-intervention in all outcome measures compared to baseline (*p* < 0.05). The DN group's MAS scores decreased from 1.7 ± 0.3 to 1.2 ± 0.2 (*p* < 0.001, *η_p_*^2^ = 0.12), GMFM-88 scores increased from 45.6% ± 6.8 to 55. 8% ± 7.1 (*p* < 0.001, *η_p_*^2^ = 0.16), and TUG times improved from 15.4 ± 2.3 s to 11.9 ± 1.8 s (*p* < 0.001, *η_p_*^2^ = 0.14). The TR group also showed significant, but smaller, improvements: MAS from 1.6 ± 0.2 to 1.4 ± 0.4 (*p* = 0.02, *η_p_*^2^ = 0.07), GMFM-88 from 44.8% ± 7.3 to 50.7% ± 8.2 (*p* = 0.01, *η_p_*^2^ = 0.09), and TUG from 15.1 ± 2.5 s to 13.7 ± 2.1 s (*p* = 0.002, *η_p_*^2^ = 0.06) (Table 1).

### 3.3 Safety-related outcomes

One mild bruise in the adductor region was reported in a female participant 24 h after treatment; it was self-limited, did not interfere with the planned physiotherapy program, and resolved within a few days.

## 4 Discussion

This randomized controlled trial was conducted to assess the adding effects of dry needling (DN) to conventional physical therapy in children with spastic cerebral palsy (CP). The two groups showed significant improvements in spasticity, gross motor function, and functional mobility over the 12-week intervention period. However, the DN group consistently reported greater improvements, with moderate differences across all outcomes. These results indicate that DN can be a clinically significant supplementary approach to conventional neurorehabilitation in this age population, in which reducing spasticity, enhancing motor activity, and increasing engagement are primary goals (27, 28, 31).

The present findings should be interpreted with caution because the study included only ambulatory children with spastic cerebral palsy classified as GMFCS levels II–III and with mild-to-moderate spasticity. Therefore, the results may not be generalized to children with more severe motor impairment, including those classified as GMFCS levels IV–V, non-ambulatory children, or children with severe cognitive impairment. Further studies are needed to determine whether the same intervention would produce similar effects in these populations.

Dry needling may reduce spasticity through peripheral and spinal mechanisms, with possible effects at higher levels of the nervous system. At the local level, needle insertion into hypertonic muscle tissue may modify abnormal muscle activity and reduce trigger point sensitivity (32). At the spinal level, it may help regulate reflex excitability and decrease exaggerated stretch responses (33). However, any proposed supraspinal effects or corticospinal reorganization (34) should be interpreted cautiously, as these mechanisms were not directly examined in children with cerebral palsy in the present study. Therefore, the present findings support a beneficial clinical effect of dry needling, while the underlying neurophysiological mechanisms remain to be clarified.

Previous randomized controlled trials of post-stroke syndrome and upper motor neuron syndrome showed short-term improvements in Modified Ashworth/Modified Tardieu scores, range of motion, and function with the use of DN alone or in combination with exercise or stretching (31, 32). Recent systematic reviews in patients who have stroke, multiple sclerosis, or traumatic brain injury have shown low-to-moderate evidence that these mechanisms provide short-term relief from focal spasticity, while highlighting the need for larger trials to establish optimal dosage and long-term effects (33, 34).

Gross motor and functional mobility depend on a series of integrated processes, including sit-to-stand, straight-line walking, turning, and controlled sitting, which are limited by lower-limb strength, spasticity, dynamic balance, and gait coordination. The latest findings by Ng and Hui-Chan (35) showed that TUG time correlates with plantarflexor spasticity, ankle weakness, gait speed, and walking endurance in patients with chronic stroke. The authors also clarified that hyperexcitable and weak distal muscles are the primary determinants of mobility impairment in those patients. Similarly, Hadi et al. (36) reported that DN reduces spasticity and modified muscle structure (spastic gastrocnemius muscles become less thick, with lower pennation angles and longer fascicles). Thus, treatment enables patients to generate greater force output at mid-range positions, decreasing co-contraction and enabling more balanced weight distribution and push-off during walking.

The relationship between spasticity reduction and improved functional mobility has been documented in several clinical studies. Ghannadi et al. (37) found that deep DN of spastic leg muscles in a randomized trial of post-stroke survivors significantly reduced MAS scores and yielded simultaneously improved TUG time, 10-m walk test speed, passive range of motion, and Barthel Index. These findings align with the present results and illustrate how decreasing segmental reflex excitability enhances gait and functional performance.

Unfortunately, the majority of the literature on DN that has been done to date in neurological rehabilitation has concentrated on adult stroke patients. There is scanty evidence that supports DN in children with CP, which mainly comes from case reports. Our results parallel those of Gallego and Mayoral (18), who reported reduced resistance to passive movement and improvements in the functional capacity of a child with hypertonia after deep DN. Simultaneously, the results of the study are consistent with those of Okonski and Dommerholt (19), who also reported increased range of motion and functional improvements in a child with spastic CP following DN with electrical stimulation. These reports, despite being encouraging, did not use control groups, randomization, or standard outcome measures and therefore limit the inferences that can be made concerning causality. The current trial therefore adds needed controlled evidence to a field dominated by descriptive reports.

Although the improvements in GMFM-88 and TUG were statistically significant, their practical relevance should also be considered. Prior research has proposed MCID estimates for GMFM-88 and TUG in children with cerebral palsy, indicating that changes of this magnitude may reflect noticeable benefits in everyday function (38–41). In our study, the DN group achieved greater improvements in GMFM-88 and completed the TUG more quickly than the control group, suggesting that the additional effect of dry needling may have functional value beyond statistical significance. Nevertheless, this interpretation should be made with caution, as MCID thresholds may vary by the child's functional level and the outcome measure used.

Safety is a critical consideration when applying invasive procedures in children. During the course of treatment in this study, DN was well tolerated with no adverse effects reported or observed in the study group. This is consistent with data on the adult neurological population, where DN is reported to be safe when provided by trained clinicians, and its side effects are often temporary, such as post-intervention soreness or minor bleeding (14, 29). Adherence to sterile technique, appropriate positioning, and the use of topical lidocaine before needle insertion likely contributed to the favorable safety profile. These strategies are invaluable in pediatric practice, where phobias about needling and heightened pain sensitivity can make treatment challenging.

The results of our report indicate that DN, when used as an adjunct therapy, produces substantial therapeutic effects when used alongside other conventional rehabilitation techniques. Introducing DN at the beginning of the session was helpful, as it minimized spasticity and maximized muscle flexibility, likely promoting outcomes from further stretching and functional training. Targeting the hamstrings, adductors, and gastrocnemius was appropriate because of their contribution to common spastic gait patterns in children with CP (42). Additionally, the treatment plan of weekly DN sessions for 12 weeks, together with three physiotherapy sessions, is both tolerable and effective. However, optimal dosing of DN still requires further study.

This study has several limitations. A single-blind methodology was used, with only the outcome assessors blinded. However, it lacked a shame needling control group to determine if performance, expectation, and placebo effects, even from guardians, occurred. The research included only ambulant children with mild-to-moderate spasticity; therefore, the results may not generalize to children with severe motor or cognitive impairments. Moreover, spasticity was assessed using the Modified Ashworth Scale, which, despite its widespread clinical use, has recognized limitations related to inter-rater reliability and its ordinal nature. Also, because outcomes were assessed only immediately after the intervention, the duration of the observed benefits remains unclear. Therefore, future studies should include medium- and long-term follow-up to determine whether these effects persist. Finally, the DN protocol used minimal needles, brief retention times, and avoided eliciting strong twitch responses to ensure safety. Still, it remains unclear if more aggressive treatment approaches would produce better results or additional complications.

## 5 Conclusion

The combination of dry needling with traditional physical therapy produced better results for spasticity reduction, motor function improvement, and mobility enhancement than traditional physical therapy alone in ambulant children with mild to moderate spastic cerebral palsy. The treatment protocol produced a significant change in the outcome measures with only brief side effects. Thus, dry needling can be considered a safe additional therapy for these children (43–49).

## Data Availability

The raw data supporting the conclusions of this article will be made available by the corresponding author, without undue reservation.
